# Potassium permanganate is an excellent alternative to osmium tetroxide in freeze-substitution

**DOI:** 10.1007/s00418-021-02070-0

**Published:** 2022-01-05

**Authors:** Martin Schauflinger, Tim Bergner, Gregor Neusser, Christine Kranz, Clarissa Read

**Affiliations:** 1 Institute of Virology, Philipps University Marburg, Hans-Meerwein-Straße 2, 35037 Marburg, Germany; 2 Electron Microscopy Core Facility, University of Missouri, 1600 East Rollins Street, Columbia, MO 65211 USA; 3 Central Facility for Electron Microscopy, Ulm University, Albert-Einstein-Allee 11, 89081 Ulm, Germany; 4 FIB Center UUlm, Institute of Analytical and Bioanalytical Chemistry, Ulm University, Albert-Einstein-Allee 11, 89081 Ulm, Germany; 5 Institute of Virology, Ulm University Medical Center, Albert-Einstein-Allee 11, 89081 Ulm, Germany

**Keywords:** Freeze-substitution, Potassium permanganate, Osmium tetroxide, Uranyl acetate, Membrane stain, Transmission electron microscopy

## Abstract

High-pressure freezing followed by freeze-substitution is a valuable method for ultrastructural analyses of resin-embedded biological samples. The visualization of lipid membranes is one of the most critical aspects of any ultrastructural study and can be especially challenging in high-pressure frozen specimens. Historically, osmium tetroxide has been the preferred fixative and staining agent for lipid-containing structures in freeze-substitution solutions. However, osmium tetroxide is not only a rare and expensive material, but also volatile and toxic. Here, we introduce the use of a combination of potassium permanganate, uranyl acetate, and water in acetone as complementing reagents during the freeze-substitution process. This mix imparts an intense en bloc stain to cellular ultrastructure and membranes, which makes poststaining superfluous and is well suited for block-face imaging. Thus, potassium permanganate can effectively replace osmium tetroxide in the freeze-substitution solution without sacrificing the quality of ultrastructural preservation.

## Introduction

Rapid cryofixation by high-pressure freezing (HPF) followed by freeze-substitution (FS) results in superior preservation of biological samples compared with conventional electron microscopy (EM) sample preparation protocols that rely on chemical fixation and dehydration of cellular structures at ambient temperatures (Dahl and Staehelin 1989; Shiurba 2001; Zechmann et al. 2007). The HPF-FS method is also compatible with subsequent embedding of the sample in methacrylate or epoxy resins. For these reasons, HPF-FS is the preferred sample preparative method for ambient-temperature transmission electron microscopy (TEM), electron tomography (ET), and block-face imaging using scanning electron microscopy [e.g., serial block-face-scanning electron microscopy (SB–FSEM) and focused ion beam scanning electron microscopy (FIB–SEM)]. During the FS process, the temperature is slowly raised, so that frozen water in the cryoimmobilized sample is gradually replaced by an organic solvent at low temperatures (Shiurba 2001). Fixatives and staining agents can be added to the solvent, which are able to react with cellular components once the temperature is high enough (Humbel and Schwarz 1989).

One of the most important and demanding tasks of biological EM is the visualization of cellular membranes. The preservation and depiction of lipid membranes is critical for the interpretation of cellular ultrastructure. However, depending on the specific biological system and methodology used, it can be challenging to achieve good membrane staining, especially in high-pressure frozen cells (Walther and Ziegler 2002). In many cases, it is necessary to treat sectioned biological specimens with heavy metal salts to achieve sufficient contrast (Reynolds 1963; Ellis 2014). However, as methods that rely on non-sectioned specimens—such as tomography and block-face imaging—become increasingly popular, it is desirable to give the bulk sample an inherently high contrast by increasing the electron density of membranes, i.e., en bloc staining (Jiménez et al. 2009; Webb and Webb 2015; Steyer et al. 2019).

Osmium tetroxide (OsO_4_) is commonly added to FS solutions because of its properties as both a fixative and stain of lipids. OsO_4_ binds to unsaturated fatty acids, and is regarded as an excellent stain for lipid-containing structures in a range of biological samples. However, OsO_4_ does not perform well in all cases, and is problematic to handle due to its toxic and volatile nature. Thus, finding osmium alternatives, as well as additives that result in an enhanced membrane staining of freeze-substituted samples, is of great interest. In the past, OsO_4_ has been used in combination with other chemical agents, such as tannic acid (Giddings 2003; Jiménez et al. 2009), imidazole (Mordhorst et al. 2018, 2019), permanganate (Heath et al. 1985), and even water (Walther and Ziegler 2002; Buser and Walther 2008) to enhance membrane staining.

We explored the use of potassium permanganate (KMnO_4_) as a reagent in FS, based on its ability to provide strong membrane contrast in conventional TEM processing and section poststaining (Luft 1956; Lawn 1960; Kaiser and Schekman 1990; Wright 2000). In this paper, we show that KMnO_4_ is an excellent en bloc stain for freeze-substituted samples, and provides high inherent electron density to cellular ultrastructure when used in combination with uranyl acetate.

## Materials and methods

Human alveolar basal epithelial cells (A549) or human foreskin fibroblasts (HFF) were grown to near confluency directly on carbon- or gold-coated sapphire disks (3 mm in diameter; Engineering Office M. Wohlwend GmbH, Switzerland). Yeast cells [*Saccharomyces cerevisiae* JD47 (Dohmen et al. 1995)] were grown in suspension medium, concentrated by centrifugation (1000 rpm, 5 min), transferred onto carbon-coated and glow-discharged sapphire disks, and let to settle for 1 min before HPF.

Samples were high-pressure frozen with a Wohlwend HPF device (either Compact 01 or 02; Engineering Office M. Wohlwend GmbH) as described previously (Schauflinger et al. 2013; Villinger et al. 2014).

The traditional FS solution consisted of acetone (VWR International GmbH, Darmstadt, Germany) with 0.2% OsO_4_ (Merck KGaA, Darmstadt, Germany), 0.2% uranyl acetate (Merck AG, Darmstadt, Germany), and 5% water.

The KMnO_4_ (reagent grade, Merck AG, Darmstadt, Germany) containing FS solutions were prepared by pipetting a small volume of freshly prepared aqueous KMnO_4_ solution on top of frozen acetone containing uranyl acetate that had been previously frozen solid by immersing cryovials upright in liquid nitrogen. It is important to note that KMnO_4_ and uranyl acetate will react with each other and precipitate if not mixed at low enough temperatures. The best results (i.e., for mammalian cells) were achieved with FS solutions of acetone containing final concentrations of 0.05% KMnO_4_, 0.1–0.2% uranyl acetate, and 2–5% water.

Quick FS was carried out in accordance with the super-quick freeze-substitution method (SQFS) described previously (McDonald and Webb 2011), with some modifications. Briefly, after HPF, the sapphire disks with the frozen cell monolayers were placed on top of previously frozen FS solution inside a cryovial. The cryovial was then closed tightly and placed into an aluminum block previously cooled to liquid nitrogen temperature. The block was turned sideways and agitated with the help of a rocker operated at 80 rpm until the block turned frost free (after about 1.6 h), at which point the samples were transferred to a fridge (ca. 4 °C) for 25 min.

Alternatively, automated FS was carried out in the chamber of an AFS2 device (Leica Microsystems GmbH, Wetzlar, Germany) by raising the temperature from −90 °C to 4 °C over a period of 17 h, followed by 30 min at 4 °C.

The freeze-substituted samples were briefly but thoroughly washed in water-free acetone, embedded in Epon resin/benzyldimethylamine (BDMA) (Embed 812, Science Services) and subsequently polymerized at 60 °C overnight. Yeast samples were embedded in Epon over 72 h on a rocker to facilitate complete resin infiltration. Ultrathin silver/grey sections were prepared, mounted on copper grids, and imaged with a JEOL JEM 1400 transmission electron microscope operated at 80 kV or 120 kV.

FIB-SEM preparation and imaging of resin-embedded samples was conducted with a Helios Nanolab 600 (Thermo Fisher Scientific, USA), exactly as described previously (Villinger et al. 2015).

## Results

**Fig. 1:**
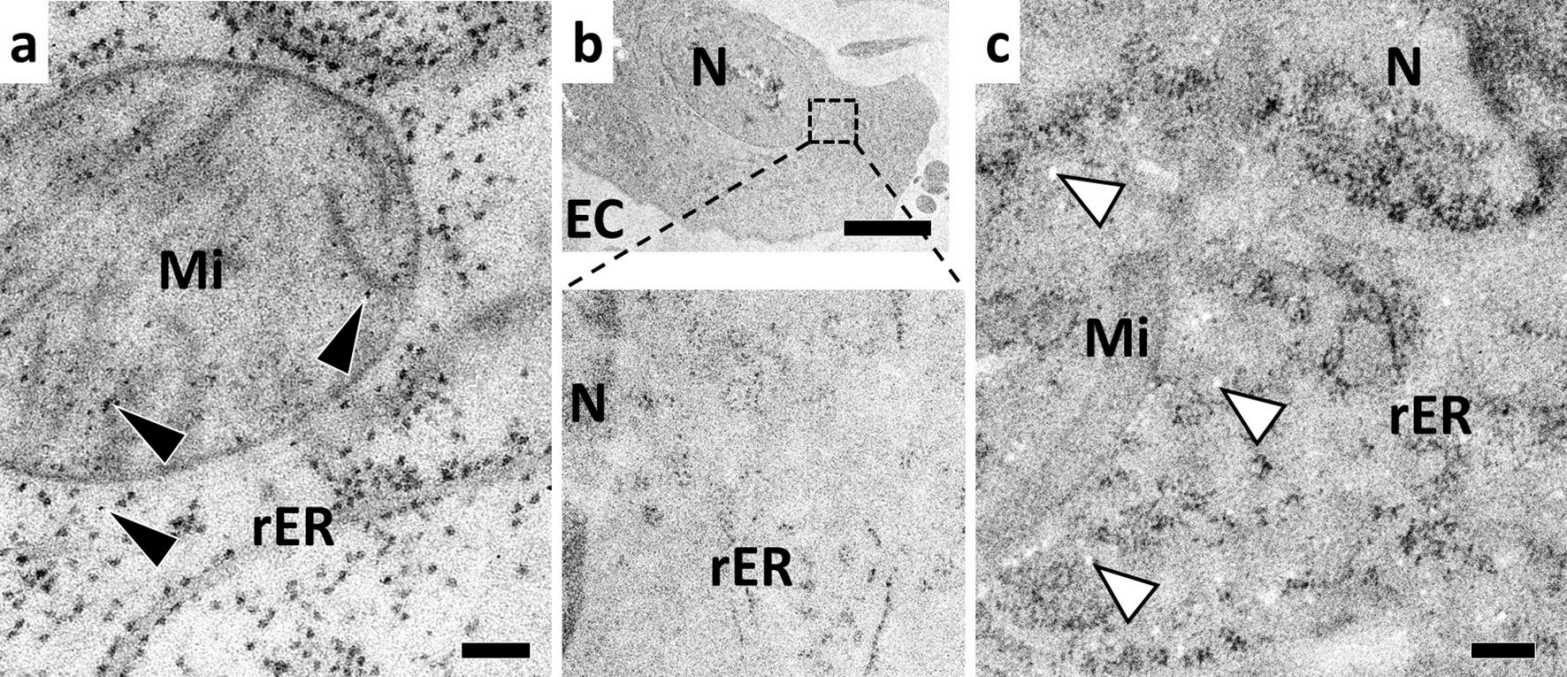
KMnO_4_ alone is not a potent membrane stain in freeze-substituted mammalian cells. A549 cells were high-pressure frozen, freeze-substituted, and embedded in Epon, and sections were observed using TEM. **a** A549 cells super-quick freeze-substituted (SQFS) with 0.2% KMnO_4_. **b** SQFS of A549 with 0.05% KMnO_4_ and 2% water. **c** HFF freeze-substituted over a period of 17 h in acetone containing 0.05% KMnO_4_ and 5% water. Black arrows depict artifactual electron dense spots, white arrows depict artifactual white spots. Bars: 100 nm (**a**), 5 µm (**b**), 1 µm (**c**). *Mi* mitochondrion, *rER* rough endoplasmic reticulum, *N* nucleus, *EC* extracellular space

Based on the ability of potassium permanganate (KMnO_4_) to provide strong membrane contrast in conventional EM processing, we explored its use as staining reagent and potential alternative to OsO_4_ in freeze-substitution (FS). A previous study used KMnO_4_ for FS of high-pressure frozen yeast, demonstrating the preservation of some ultrastructural features and membranes of HM20 embedded yeast cells (Giddings 2003). In our tests, the incorporation of KMnO_4_ to the FS solution without the addition of other reagents did not provide consistent results nor sufficient staining in cultured mammalian cells (Fig. 1). Usually, the addition of KMnO_4_ to acetone resulted in a relatively weak staining of the nuclear lamina, cytoskeletal elements, and the mitochondrial matrix, while ribosomes were clearly visible. Occasionally, we observed a weak staining of some membranes and an increased electron density of the ER lumen (Fig. 1a). Most of the time, the contrast was extremely weak and membranes were either not or only barely visible, even with extended FS times or the addition of water (Fig. 1b, c). Furthermore, the use of KMnO_4_ under these conditions could lead to the formation of artifacts such as electron dense deposits (Fig. 1a) or white spots (Fig. 1c). Similar problems were observed when the concentration of KMnO_4_ was too high, which is why we used working concentrations of well below 0.1% (w/v) KMnO_4_ in the following experiments.

**Fig. 2:**
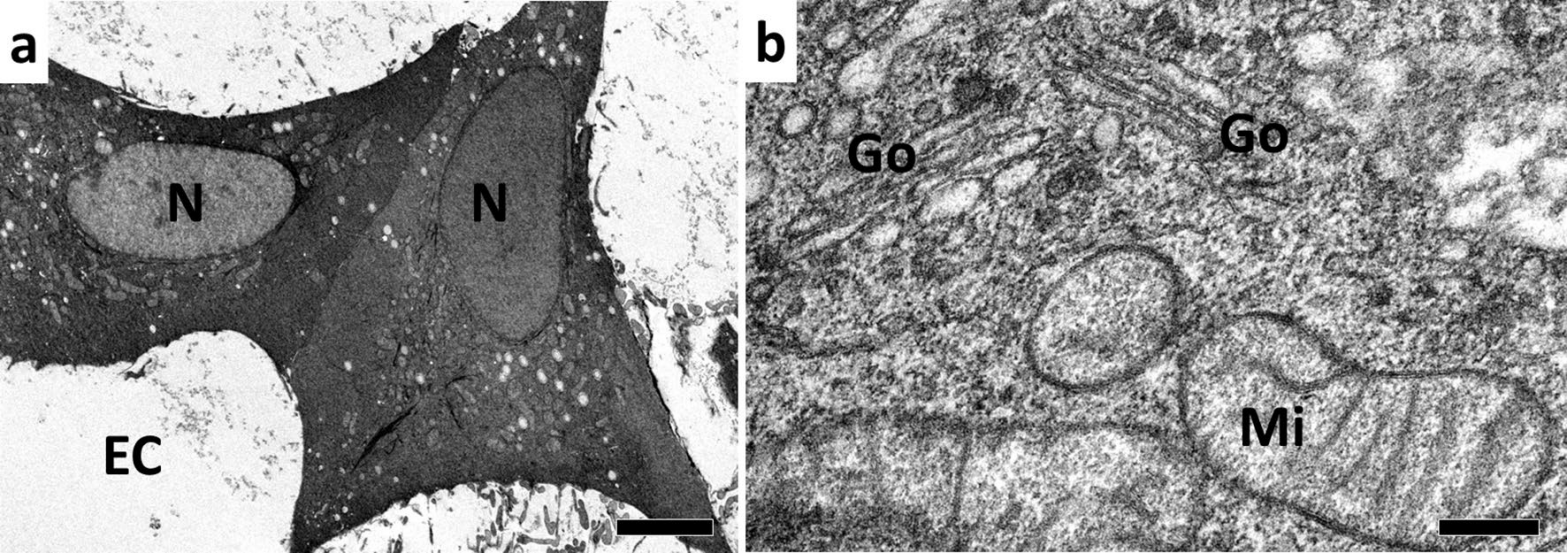
KMnO_4_ in combination with uranyl acetate results in very intense staining of cellular ultrastructure. A549 cells were high-pressure frozen followed by SQFS with 0.05% KMnO_4_ and 0.2% uranyl acetate, embedded in Epon, and sections were observed using TEM. **a** Overview, **b** detail. Bars: 5 µm (**a**), 200 nm (**b**). (*N* nucleus, *EC* extracellular space, *Go* Golgi apparatus, *Mi* mitochondrion)

Since KMnO_4_ by itself was not sufficient as a fixative or staining agent, we explored the supplementation of additional chemical agents to the FS solution. We found that the combination of KMnO_4_ and uranyl acetate returned promising results in freeze-substituted mammalian cells (Fig. 2). To circumvent the precipitation of KMnO_4_ and uranyl acetate, which happened upon mixing both agents together at room temperature, we exploited the fact that KMnO_4_ and uranyl acetate did not react with each other at low temperatures. Thus, one part of the solution was frozen first (i.e., uranyl acetate in acetone), and only then was the KMnO_4_ solution added. Low concentrations of KMnO_4_ were sufficient to produce very strong staining when uranyl acetate was included in the FS solution, and the membranes showed very clearly the railroad track morphology of the lipid bilayer (Fig. 2b). However, the cytoplasm appeared relatively dark, which made it difficult to distinguish individual features, such as organelles or cytoskeletal structures.

**Fig. 3:**
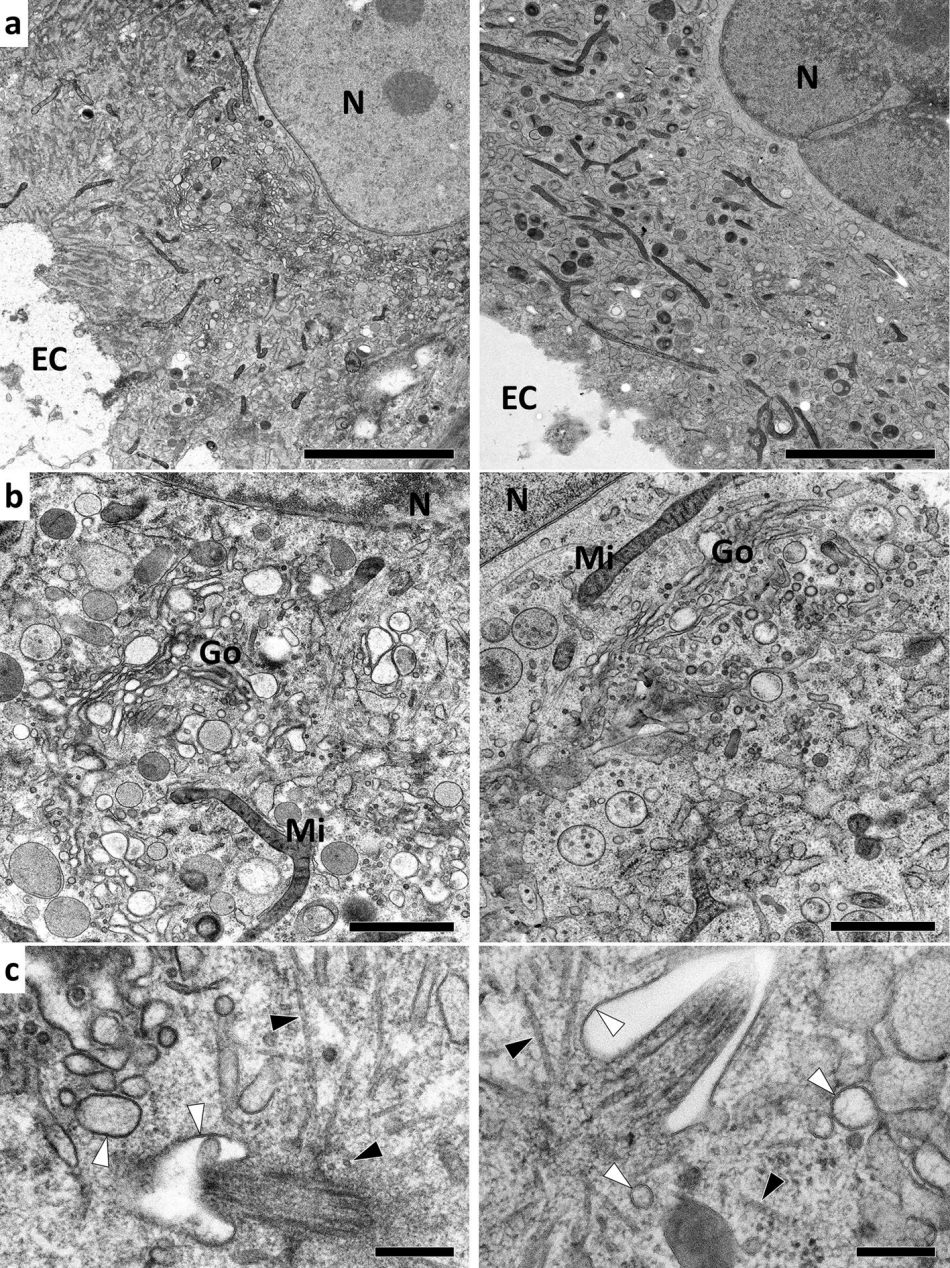
FS with a combination of KMnO_4_, uranyl acetate, and water (left panel) results in excellent preservation and staining of the cellular ultrastructure, similar to a standard FS solution that contains OsO_4_, uranyl acetate, and water (right panel). HFF were high-pressure frozen and freeze-substituted over 17 h in acetone containing 0.2% uranyl acetate, 5% water, and additionally either 0.05% KMnO_4_ (left panel) or 0.2% OsO_4_ (right panel). The cells were then embedded in Epon, and ultrathin sections were observed on TEM at 80 kV. **a** Low magnification overview, **b** perinuclear region, **c** higher magnification of the centrosomal region showing details of lipid bilayers (white arrows) and microtubules (black arrows). Bars: 5 µm (**a**), 1 µm (**b**), 250 nm (**c**). *Mi* mitochondrion, *N* nucleus, *EC* extracellular space, *Go* Golgi apparatus

Interestingly, the addition of a small amount of water to the FS solution resulted in a much less intense staining of the cytoplasm, seemingly without affecting the strong staining of other ultrastructural features, such as the lipid bilayers and microtubules (Fig. 3). This led to increased visibility of individual organelles and cytoskeletal elements, which allowed detailed analysis of the cellular ultrastructure. The combination of KMnO_4_, uranyl acetate, and water described here worked equally well with both the more conventional FS times (> 16 h) and the very short FS schedules described more recently as super-quick freeze-substitution (SQFS) (McDonald and Webb 2011). To achieve sufficient staining, it appeared beneficial to incubate the samples at a temperature slightly above 0 °C for at least a few minutes at the end of each FS run. On the other hand, it was critical not to let the sample reach room temperature, because this usually resulted in artifacts such as overstaining and degradation of the fine cellular ultrastructure.

For comparison, we simultaneously processed high-pressure frozen HFF cells in a standard FS solution containing OsO_4_. The results achieved with the FS mixture of 0.05% KMnO_4_, 0.2% uranyl acetate, and 5% water in acetone (Fig. 3, left panel) were almost indistinguishable from the FS solution used routinely in the Ulm laboratory, which consists of 0.2% OsO_4_, 0.2% uranyl acetate, and 5% water in acetone (Fig. 3, right panel). In comparison, the KMnO_4_ treated cells exhibited a stronger staining intensity, which was especially noticeable on the TEM viewing screen, where it was possible to easily distinguish features of the KMnO_4_ treated samples by eye, while OsO_4_ treatment resulted in a relatively weak contrast.

**Fig. 4:**
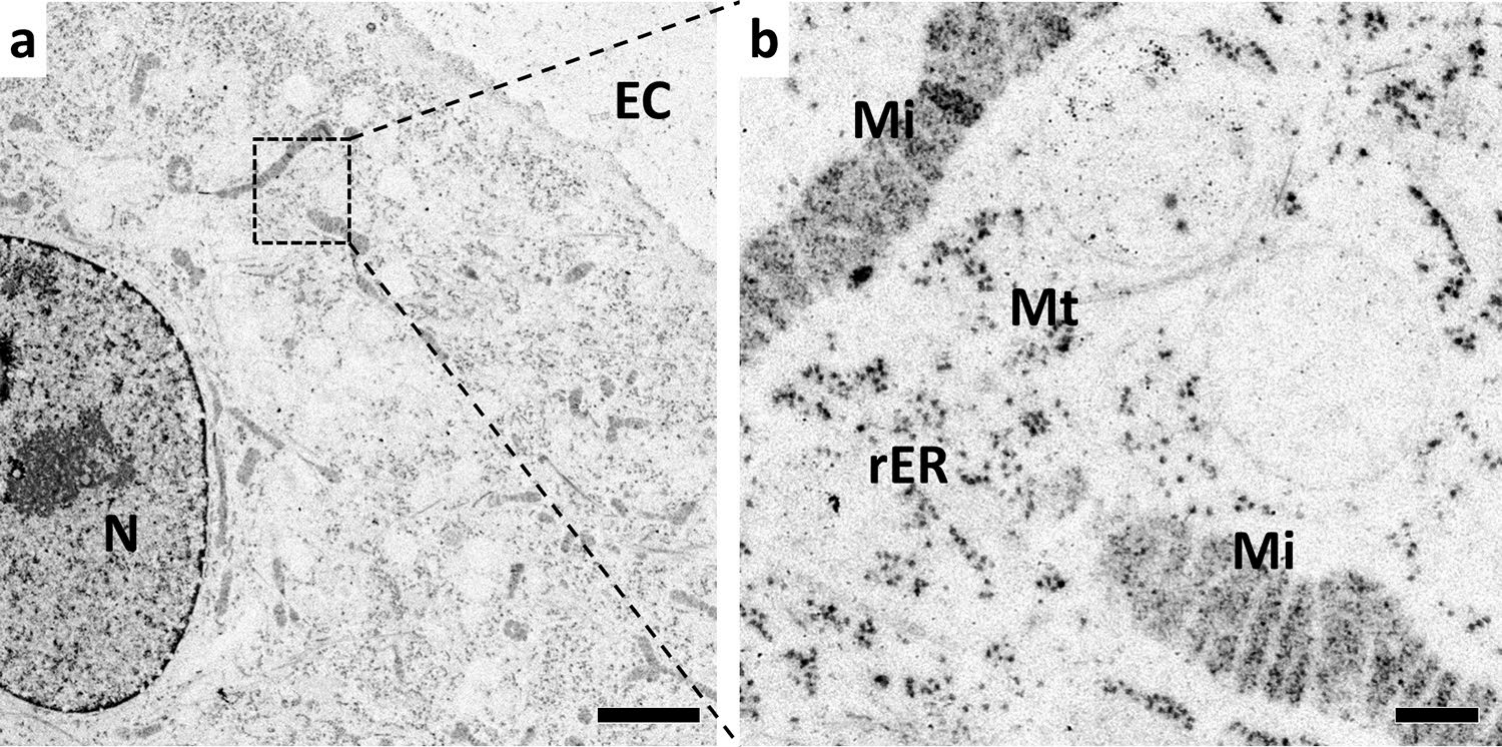
Uranyl acetate alone is not a sufficient membrane staining agent. A549 cells were high-pressure frozen, SQFS with acetone containing 0.2% uranyl acetate and 2% water, embedded in Epon, and sections were observed using TEM. **a** Overview, **b** detail. Bars: 2 µm (**a**), 200 nm (**b**). *N* nucleus, *EC* extracellular space, *rER* rough endoplasmic reticulum, *Mi* mitochondrion, *Mt* microtubules

The interaction between KMnO_4_ and uranyl acetate appeared to be responsible for the excellent staining of membranes, which can be demonstrated by observing sections after using uranyl acetate as the sole ingredient with acetone in the FS solution (Fig. 4). While uranyl acetate stained structures rich in nucleic acids, such as ribosomes and cell nuclei, as well as cytoskeletal structures, it was not suitable as a membrane stain. Since neither KMnO_4_ nor uranyl acetate alone stained membranes, a complementary action of both agents likely caused the well-defined membrane staining observed in the presence of KMnO_4_ and uranyl acetate together.

**Fig. 5:**
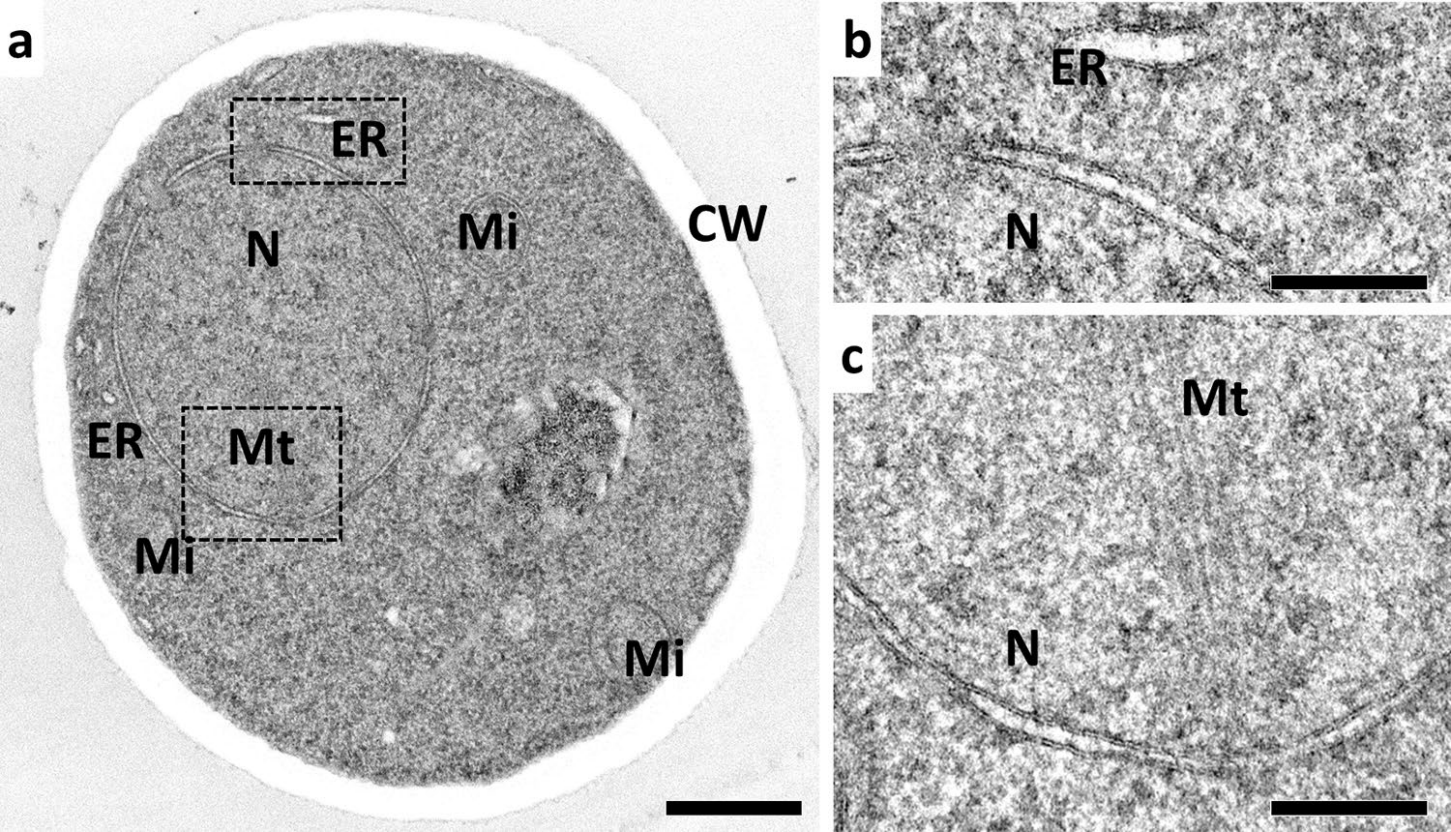
FS with KMnO_4_, uranyl acetate, and water provides good visualization of membranes and microtubules of a yeast cell. A suspension of *S. cerevisiae* was high-pressure frozen, freeze-substituted over a period of 17 h in acetone containing 0.05% KMnO4, 0.2% uranyl acetate, and 5% water, and embedded in Epon, and sections were observed using TEM at 120 kV. Bars: 500 nm (**a**), 200 nm (**b**, **c**). *ER* endoplasmic reticulum, *N* nucleus, *Mi* mitochondrion, *CW* cell wall, *Mt* microtubules of the spindle pole body

Since KMnO_4_ has historically been used especially for TEM preparation of yeast, we also explored its applicability on high-pressure frozen samples of *S. cerevisiae* (Fig. 5). We achieved decent staining of the cellular components when using a combination of 0.05% KMnO_4_, 0.2% uranyl acetate, and 5% water, and could observe well-defined membranes as well as microtubules. These results were comparable to FS with OsO_4_ (not shown), and indicate that FS with a combination of KMnO_4_, uranyl acetate, and water could likely be adapted for ultrastructural analyses of various (micro-) biological samples.

**Fig. 6:**
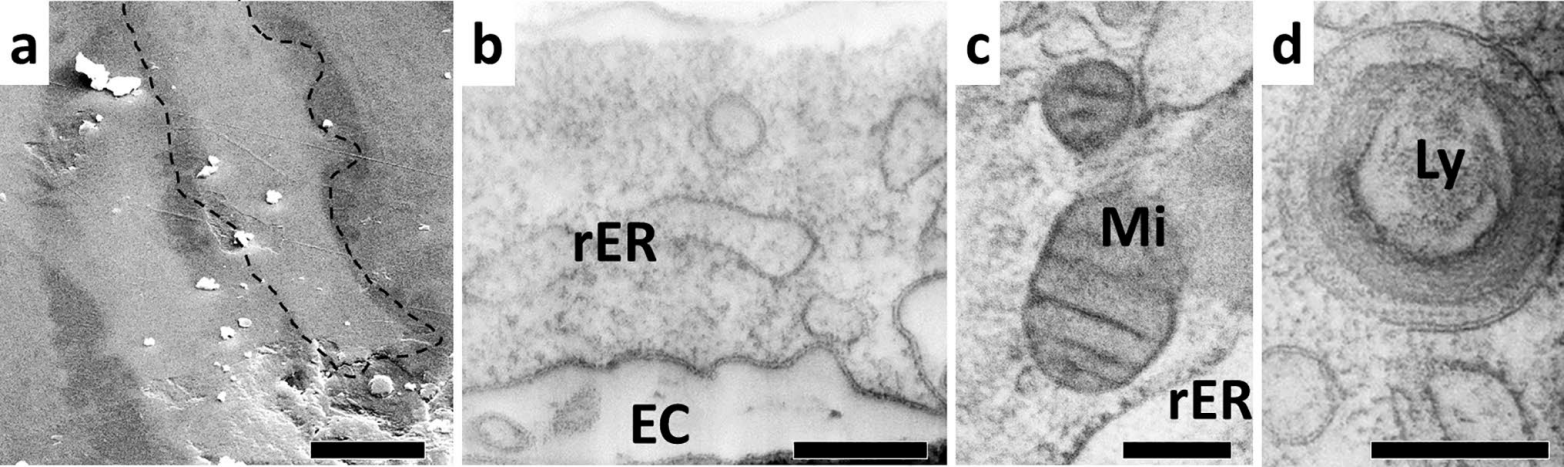
FS with KMnO_4_ and uranyl acetate is suitable for FIB-SEM analyses. HFF were high-pressure frozen, freeze-substituted over a period of 17 h in acetone containing 0.05% KMnO_4_, 0.2% uranyl acetate, and 5% water, and embedded in Epon. The resin-embedded sample was mounted on a stub, and then a block face was created and imaged by FIB-SEM. **a** View onto the embedded cells at 15 kV acceleration voltage before milling into the outlined cell. **b**–**d** Views from the block face after milling, taken by detection of secondary electrons at 5 kV (inverted contrast). Bars: 15 µm (**a**), 250 nm (**b**–**d**). *rER* rough endoplasmic reticulum, *EC* extracellular space, *Mi* mitochondrion, *Ly* lysosome

Finally, we wanted to determine if FS with a combination of KMnO_4_, uranyl acetate, and water was suited for use in block-face imaging, especially for focused ion beam scanning electron microscopy (FIB–SEM). This method has become quite popular with biologists, but as there is no possibility of post-section staining, it requires sufficient en bloc stain. Image formation in SEM differs from TEM. While the contrast in TEM is created by the transmission of electrons through a thin sample, the SEM image is formed by detecting secondary or backscattered electrons originating from the block face. Thus, the conductivity of the investigated block face needs to be high enough to prevent sample charging during SEM acquisition. Remarkably, in high-pressure frozen mammalian cells, FS with a combination of KMnO_4_, uranyl acetate, and water produced strong en bloc staining, which was suitable for FIB-SEM analyses of resin-embedded samples (Fig. 6). The cells were sufficiently stained to be recognizable under the resin surface at 15 kV acceleration voltage, which is important for localizing cells of interest (Fig. 6a). Slice and view imaging at 5 kV, and detection of the secondary electron signal with the through-the-lens detector resulted in images with TEM-like resolution (Fig. 6b–d), comparable to the quality achieved with FS solutions containing OsO_4_, uranyl acetate, and water (Villinger et al. 2012).

## Discussion

This study was born out of the necessity to find a suitable replacement for OsO_4_ in the laboratory of the EM Core Facility of the University of Missouri. In this paper, we show that KMnO_4_ in combination with uranyl acetate in the FS solution can effectively replace OsO_4_ as a fixative and staining agent in different cultured cells. The addition of water to the FS solution lowered the overall staining intensity and thus enhanced the appearance of the cellular ultrastructure. In general, the staining of biological features appeared to be stronger when KMnO_4_ instead of OsO_4_ was used, which for most purposes should render poststaining of sections unnecessary.

Aqueous KMnO_4_ was one of the first fixatives to be used for EM preparations (Luft 1956). It still has its place as postfixative, with or without additional aldehyde fixation, because of its good membrane preserving properties, especially for the preparation of yeast cells (Wright 2000; Heiman and Walter 2000). However, permanganate was not seen as a general alternative to OsO_4_, although membranes were preserved and contrasted well. Aqueous permanganate solutions did not preserve cytoskeletal and other nonlipid structures; it altered the morphology of some organelles and degraded fine ultrastructure when employed as fixative at ambient temperature (Luft 1956; Frankl et al. 2015). These detrimental effects are probably from the strong oxidizing property of permanganate that affects nonmembranous structures (Hopwood 1969; Hayat 1993; Wright 2000). There could be a number of reasons for the prevention or mitigation of detrimental effects by KMnO_4_ in FS, for instance, the reduced reactivity of permanganate at low temperatures and low concentrations of water, as well as the presence of uranyl acetate, which seems to serve as a reaction partner of permanganate. The latter could be responsible for a further decrease in the concentration of the already relatively low amounts of KMnO_4_ in the FS solution, thus limiting the chance of detrimental reactions with the sample material. Consistently, we and others have noticed harmful effects of KMnO_4_ exacerbated by increased working concentrations of KMnO_4_, as well as by higher temperatures at the end of the FS run (Giddings 2003).

Based on its ability to provide strong membrane contrast in conventional EM processing and its use for section poststaining, KMnO_4_ had previously been explored as reagent in FS of high-pressure frozen samples, with somewhat underwhelming results (Giddings 2003; Hess 2003). One study recommended a systematic analysis of the optimum concentration, temperature, and time parameters for FS with KMnO_4_ in acetone (Giddings 2003). However, as we demonstrate in the present paper, the true potential of KMnO_4_ as a staining agent appears to be unlocked by its interaction with uranyl acetate. Uranyl acetate has been in use for a long time as both a fixative and heavy metal stain in TEM analyses of biological samples. However, uranyl acetate alone is not a sufficient membrane staining agent, and evidence suggests that the previously reported effect of uranyl acetate on the marked improvement in the preservation of cell membranes is due to a fixating rather than a contrasting action (Silva et al. 1968, 1971). On the other hand, KMnO_4_ has been noted as an excellent reagent for revealing and studying membrane structures within the cell, but was considered to act as a stain rather than a true fixative (Bradbury and Meek 1960; Wright 2000). Thus, the excellent membrane staining property of the KMnO_4_ in combination with uranyl acetate in the FS solution reported here is most likely mediated by the complementary reaction of KMnO_4_ with (membrane-bound) uranyl acetate. The fact that KMnO_4_ and uranyl acetate precipitate when mixed together at ambient temperature is evidence for an interaction between these reagents.

Initially, the addition of up to 10% of water to the FS solution was introduced as a way to improve membrane staining in combination with OsO_4,_, even though the precise mechanism of this action is not entirely clear (Walther and Ziegler 2002; Buser and Walther 2008). Water-free FS with KMnO_4_ and uranyl acetate imparts intense staining to the membrane bilayers, but can also darken the cytoplasm considerably. Addition of water to this KMnO_4_ and uranyl acetate containing FS solution causes a marked decrease in the staining intensity of the cytoplasm without affecting the membrane staining, which allows an easier differentiation of individual structures and organelles. Although the processes underlying the observed effect are not clarified, it appears as if the added water affects the interactions between KMnO_4_, uranyl acetate, and/or the sample materials. We could observe good results for mammalian cells upon adding 2–5% of water to the FS solutions containing KMnO_4_ and uranyl acetate. The optimal balance of these ingredients in FS solutions will likely depend on the respective biological sample and question of interest, and thus may require more empirical testing and optimization for different specimens and methods.

We could successfully demonstrate the high resolution block-face imaging of KMnO_4_ stained FIB-SEM samples by detecting the secondary electron signal, in line with our previous work for OsO_4_ treated samples (Villinger et al. 2012). One advantage of secondary electron block-face images are that the measurable secondary electrons are produced close to the surface, which is beneficial in achieving a high resolution. Backscattered electron imaging, on the other hand, usually provides a higher contrast at the expense of resolution (Winter et al. 2009). One would expect a lower backscattered electron signal when osmium (atomic weight 190) is exchanged with manganese (atomic weight 55), with a considerable amount of signal being contributed by scattering at the uranium (atomic weight 238). Thus we recommend using secondary electron detection for block-face imaging of KMnO_4_ stained samples. Taken together, our data demonstrates that OsO_4_ in the FS solution can be replaced by a low concentration of KMnO_4_ for ultrastructural investigations of high-pressure frozen, freeze-substituted, resin-embedded cells. A replacement of OsO_4_ by KMnO_4_ has several advantages, while the achieved ultrastructural preservation and staining is at least equivalent. The use of KMnO_4_ contributes to workplace and environmental safety, since OsO_4_ crystals and solutions are volatile, toxic, and must be handled and disposed of very carefully. Furthermore, KMnO_4_ is abundant and much cheaper compared with the relatively rare and expensive OsO_4_. Finally, KMnO_4_ can serve as an alternative in cases where OsO_4_ does not perform well.

## Data Availability

The data generated during the current study are available from the corresponding author on reasonable request.
